# Blood cell transcript levels in 5-year-old children as potential markers of breastfeeding effects in those small for gestational age at birth

**DOI:** 10.1186/s12967-019-1896-1

**Published:** 2019-05-07

**Authors:** Julio Alvarez-Pitti, Maria Amparo Ros-Forés, Ana Bayo-Pérez, Mariona Palou, Empar Lurbe, Andreu Palou, Catalina Picó

**Affiliations:** 1 Pediatric Department, Consorcio Hospital General, University of Valencia, Valencia, Spain; 2 CIBER Fisiopatología Obesidad y Nutrición (CIBEROBN), Instituto de Salud Carlos III, Madrid, Spain; 3 INCLIVA Biomedical Research Institute, Hospital Clínico. University of Valencia, Valencia, Spain; 4 Laboratory of Molecular Biology, Nutrition and Biotechnology (Nutrigenomics), University of the Balearic Islands (UIB), Palma de Mallorca, Spain; 5 Instituto de Investigación Sanitaria Illes Balears (IdISBa), Palma de Mallorca, Spain

**Keywords:** Breast milk, Infant formula, Biomarkers, Metabolic programming, Cardiometabolic risk factors

## Abstract

**Background:**

Nutrition of the newborn during the early postnatal period seems to be of capital importance and there is clinical evidence showing the protective effect of breastfeeding compared with formula feeding on childhood obesity and its comorbidities. Infants born small for gestation age may be more sensitive to the type of feeding during lactation. Here, we aimed to analyze the impact of birth weight and the type of infant feeding on the expression levels in peripheral blood cells of selected candidate genes involved in energy homeostasis in 5-year-old children, to find out potential early biomarkers of metabolic programming effects during this period of metabolic plasticity.

**Methods:**

Forty subjects were recruited at birth and divided in four groups according to birth weight (adequate or small for gestational age) and type of infant feeding (breastfeeding or formula feeding). They were followed from birth to the age of 5 years.

**Results:**

At 5 years, no significant differences regarding anthropometric parameters were found between groups, and all children had normal biochemical values. Expression levels of *UCP2* and *MC4R* in peripheral blood cells were lower and higher, respectively, in formula feeding children compared with breastfeeding ones (*P *= 0.002 and *P *= 0.064, two-way ANOVA). Differences were more marked and significant by Student’s *t* test in small for gestation age children (*P *< 0.001 and *P* = 0.017, respectively). Transcript levels of *FASN* and *FTO* in peripheral blood cells were also different according to the type of infant feeding, but only in small for gestation age children.

**Conclusions:**

Altogether, these results suggest that small for gestation age infants are more sensitive to the type of feeding during lactation, and transcript levels of particular genes in peripheral blood cells, especially the *MC4R*/*UCP2* mRNA ratio, may precisely reflect these effects in the absence of clear differences in phenotypic traits.

## Background

Intrauterine and early life events are thought to be very important in the development of chronic disorders, contributing to the developmental origins of cardiometabolic disease mainly in adults [1]. Much evidence supports the fact that low birth weight children have an increased risk to develop cardiovascular illnesses compared with normal weight ones [2]. Poor growth in utero contributes to insulin resistance, significant increased risk for type 2 diabetes mellitus, obesity, hypertension, dyslipidemia, and coronary heart disease [2, 3]. The time immediately before and after birth may be a sensitive period related to programming cardiometabolic risk [4]. Therefore, a critical window of opportunity to modify programming may exist during pregnancy and throughout the first years of life. Conditions or dietary interventions during the lactation period may even potentially reverse metabolic malprogramming due to adverse in utero conditions, as demonstrated in animal models of maternal undernutrition [5, 6].

In this regard, nutrition of the newborn during the early postnatal period seems to be of capital importance [7]. Different lines of clinical evidence show the protective effect of breastfeeding against childhood obesity and its comorbidities [8, 9]. Several theories have been proposed to explain this protective effect, including behavioral and nutritional explanations [10], such as differences in milk energy density and macronutrient composition [11], the impact of breastfeeding on microbiota composition [12], or the implication of specific bioactive compounds [13], particularly those that occur naturally in human breast milk and are absent in infant formula, such as leptin [14–17]. It is likely that beneficial effects of breastfeeding are particularly relevant in low birth weight infants [18].

To be able to predict the increased risk of metabolic-syndrome related alterations due to early programming events is a challenge. This may allow the application of strategies at a time when there are more opportunities to prevent the increased risk, and before phenotypic alterations become evident. Thus, it must be helpful to have reliable, early biomarkers of increased propensity to these alterations and sensitive to nutritional interventions at early life.

Peripheral blood cells, considering whole peripheral blood (PBC) or a subpopulation of white blood cells composed basically by lymphocytes and monocytes, the so-called peripheral blood mononuclear cells (PBMC), are easily accessible from blood samples, and they have been proposed as a useful source of markers to potentially predict some aspects of the pathological and physiological state of the organism [19, 20]. We have previously described the presence of low expression levels of energy metabolism-related genes, such as *INSR*, *CPT1A* and *FASN,* in PBC of obese children with high plasma triglyceride levels, whereas children with high expression levels displayed plasma levels of TG similar to normal-weight children [21]. In the case of *CPT1A,* low expression levels were also related to higher HOMA index in obese children [21]. Therefore, expression levels of these genes were proposed as potential biomarkers of the metabolic status of obese children as they are indicative of the risk for the insulin-resistant or dyslipidemic state associated with obesity. Moreover, in a previous study we also found a relationship between breastfeeding and an increased expression of some of these protective biomarkers; breastfed children displayed higher expression levels of *INSR* and *FASN* in PBC compared with formula-fed subjects [22]. Determination of expression levels of these and other potential candidate genes related with energy homeostasis in children born with low or adequate weight may be of interest, particularly as they may reflect the beneficial effects of breastfeeding.

Therefore, the objective of the present study was to analyze the impact of birth weight and the type of feeding during infancy (breastfeeding or formula feeding) on the expression levels in PBC of selected candidate genes involved in energy homeostasis (*CPT1*, *FAS, FTO, INSR*, *LEPR*, *MC4R*, and *UCP2*) in a subset of children followed from birth to the age of 5 years to find out potential biomarkers of metabolic programming effects during this period of metabolic plasticity.

## Methods

### Participants

Subjects involved in this study were a subset of 40 infants from the COMETA cohort and born in the *Hospital General Universitario de Valencia*, Spain [23]. Subjects invited to participate were newborns born at term (gestational age ≥ 37 weeks) after uncomplicated pregnancies and in the absence of perinatal illness. Exclusion criteria were multiple gestations, cesarean section or plan to move out of the area after delivery. The subjects included in the present study (males and females) were randomly selected at birth, according to body weight (BW) for gestational age (appropriate or small) and the planned type of infant feeding (breast feeding or formula feeding). Gestational age at birth was ascertained according to the method of Ballard et al. [24]. Infants that were below the 10th percentile for gestational age and sex were considered small for gestational age (SGA), and those between the 10th and 90th percentile were considered appropriate for gestational age (AGA) [25]. The general characteristics of gestation and delivery were obtained from routine obstetric records. The type of feeding was assessed during the first year of life. Infants that were exclusively fed with formula were classified as formula-fed group (FF). Those that were breastfed for at least 3 months, even when combined with formula, were classified as breastfed (BF), as other studies have described significant differences in the prevalence of obesity in children who were never breastfed compared with those ever breastfed, including short periods of breastfeeding [8, 22, 26].

At birth, all parents gave permission for their children to participate in the study and informed consent was obtained. The Committee for the Protection of Human Subjects of the Hospital General approved the study according to the Second declaration of Helsinki (2013).

### Anthropometric and biochemical measurements

At birth, nude body weight was obtained by using a Seca 728 scale in duplicate with both measures having to be within 10 g; in the event they fell outside of these predefined parameters, a third measure was obtained with the 2 closest values averaged. Crown-to-heel length at birth was obtained by using a Seca 416 infantometer in duplicate with both measures having to be within 0.1 cm; in the event they fell outside of these predefined parameters, a third measure was obtained with the 2 closest averaged values.

At 5 years, body weight was recorded to the nearest 0.1 kg using a standard beam balance scale with the subjects wearing light indoor clothing and no shoes. Height was recorded to the nearest 0.5 cm using a standardized wall-mounted height board. Body mass index (BMI) and the corresponding standard deviation (SD) were calculated, with BMI being the weight in kilograms divided by the square of the height in meters. BMI z score (BMIz) was calculated by expressing a child’s BMI relative to children in the World Health Organization (WHO) growth charts on the basis of the previously described method [27]. Values of L (lambda), M (mu) and S (sigma) varied according to a child’s sex and age. Subjects with a BMI z score ranging from 1 to 2 were defined as being overweight. Obesity was defined as having a BMI +2SD value, following WHO recommendations [28].

At 5 years of age, a metabolic assessment was performed under fasting conditions in the early morning. Peripheral blood samples were obtained to measure glucose by the glucose oxidase method (Beckman Glucose Analyzer, Beckman Instruments, Fullerton, CA), insulin (Pharmacia Insulin RIA kit; Uppsala, Sweden), lipid profile, and uric acid. The homeostatic model assessment (HOMA) index was calculated by dividing the product of insulin (microunits per milliliter) and glucose (millimoles per liter) by 22.5 [29].

### Casual blood pressure measurements

At birth (on the second day of life) and at 5 years, blood pressure (BP) measurements were performed. Nurses measured the BP three consecutive times using a Dinamap (Pro Care, GE Medical Systems Information Technologies, Inc, Milwaukee, WI) oscillometric recorder [30]. The mean of the three measurements was taken as the casual BP. For measurements made at birth, the subjects were in the supine position or in the care provider’s lap, in a state of quiet sleep or quiet wakening. For measurements at 5 years, the child was seated. The appropriate cuff, placed on the left arm, was selected according to the length of the upper arm of each subject; the cuff extended completely around the arm and width of the bladder covered at least two-thirds of the upper arm [31].

### Blood sampling and processing for gene expression analysis

At 5 years of age, a total of 2.5 ml peripheral blood was collected from each participant under fasting conditions into PAX gene vacutainer tubes (QIAGEN, Hilden, Germany) via antecubital fossa venipuncture, following the manufacturer’s instructions (QIAGEN). This system allows collection and immediate stabilization of intracellular RNA of total blood cells without further manipulations, facilitating standardisation and reproducibility. This makes it an attractive approach in human studies [21].

Total RNA was isolated using the PAX gene blood RNA kit according to the manufacturer’s instructions (QIAGEN). RNA quality and purity were analysed by spectrophotometry using the Nanodrop ND-1000, and RNA integrity was confirmed using agarose gel electrophoresis.

### Real-time quantitative RT-PCR analysis

Expression levels of selected genes were determined by quantitative RT-PCR analysis. Genes analysed were: carnitine palmitoyl transferase 1 alpha (*CPT1A)*, fatty acid synthase (*FASN),* alpha-Ketoglutarate dependent dioxygenase (*FTO)*, *insulin receptor (INSR)*, leptin receptor, long-form (*LEPR)*, melanocortin 4 receptor (*MC4R)*, and uncoupling protein 2 (*UCP2).* Selection of genes was based both on previous studies showing changes in their expression levels in PBC of children according to type of infant feeding, body weight, and/or metabolic status (the most representative of them), and their relevance in energy homeostasis and obesity predisposition. Specifically, *FASN*, *INSR* and *UCP2* were found to be differently expressed in PBC between breastfed and formula fed children [22]; expression levels of *CPT1A*, *INSR*, FASN, LEPR were related to insulin-resistant or dyslipidemic state of children [21]; and expression levels of MC4R were described to be associated with BMI z-score, and body fat percentage [32]. *FTO* was also analysed because of the relevance of gene variants on obesity predisposition [33, 34]. All primers were obtained from Sigma Genosys (Sigma-Aldrich Quimica SA, Madrid, Spain).

Quantitative RT-PCR was performed as previously described [35]. The threshold cycle (Ct) was calculated using the instrument’s software (StepOne Software version 2.0), and the relative expression ratio of a target gene was calculated based on the corresponding real-time PCR efficiency and Ct deviation of an unknown sample vs. mean Ct of all samples and expressed in comparison to a reference gene [36]. *TRIM27* (*Homo sapiens tripartite motif containing 27*) was chosen as reference gene because expression levels were stable across all groups. *TRIM27* has been previously used as reference gene in PBC samples in children [37].

### Statistical analysis

Data are expressed as mean ± SD. Differences among the groups of study were assessed using two-way ANOVA. Student’s t test was used to compare individual means.

Multiple linear regression analysis, using anthropometric, BP values and metabolic parameters as dependent variables and expression of the different genes, as independent variables, was calculated.

## Results

### Characteristics of study subjects

Of the 50 subjects who accepted the invitation to participate in the present study, 10 did not have follow-up data. The remaining forty subjects (21 males and 19 females), who fulfilled the inclusion criteria and had their data recorded at follow up, were included in the study.

General characteristics of the sample, divided into four groups in terms of weight (AGA and SGA) and type of feeding at birth (BF and FF) are shown in Table 1. At birth, mean gestational age was 39.1 ± 1.4 weeks, and the average BW was 2909 ± 561 g. SGA infants had lower values of systolic BP than AGA children (*P *= 0.041, two-way ANOVA). At 5 years of age, 40% of all subjects were overweight or obese, but no significant differences were found in the distribution among groups (data not shown). The high prevalence of obesity in the study population could be seen as not representative of the general population. Nevertheless, the prevalence of overweight and obesity in the AGA group was similar to the average prevalence in Spanish children [38]. No significant differences regarding anthropometric parameters and blood pressure values were found between groups.

**Table 1:** General characteristics of the sample grouped by birth weight and type of infant feeding

Variable (mean ± SD)	AGA (n = 21)		SGA (n = 19)		ANOVA
	BF (n = 8)	FF (n = 13)	BF (n = 10)	FF (n = 9)	
Subjects (total%)	20.0	32.5	25.0	22.5	
Sex m/f	5/3	8/5	3/7	5/4	
At birth					
Weight (g)	3439 ± 236	3335 ± 330	2390 ± 248^w^	2401 ± 225^w^	W (*P *< 0.001)
Height (cm)	51.0 ± 2.5	50.0 ± 2.0	46.0 ± 1.2^w^	46.5 ± 2.2b^w^	W (*P *< 0.001)
SBP (mmHg)	78 ± 9	69 ± 8^f^	66 ± 10b^w^	68 ± 10	W (*P* = 0.041)
DBP (mmHg)	45 ± 7	42 ± 9	40 ± 10	37 ± 8	ns
HR (bpm)	124 ± 8	126 ± 19	122 ± 21	129 ± 14	ns
At 5 year old (mean age)	4.7 ± 0.7	5.2 ± 0.6	5.2 ± 0.9	5.1 ± 0.6	ns
Weight (kg)	23.0 ± 6.2	21.3 ± 4.2	20.0 ± 3.4	20.2 ± 3.2	ns
Height (cm)	112 ± 9	112 ± 6	110 ± 5	112 ± 5	ns
BMI (kg/m^2^)	18.0 ± 2.3	16.9 ± 3.0	16.4 ± 2.2	15.9 ± 1.8	ns
BMI percentile	86 ± 14	65 ± 35	64 ± 33^w^	58 ± 27	ns
BMI-Z score	1.59 ± 1.12	0.86 ± 1.86	0.65 ± 1.28^w^	0.36 ± 1.02	ns
SBP (mmHg)	97 ± 5	91 ± 7	96 ± 7	95 ± 10	ns
DBP (mmHg)	53 ± 5	54 ± 5	58 ± 4	56 ± 11	ns
HR (bpm)	96 ± 6	88 ± 29	99 ± 13	95 ± 8	ns
Glucose (mg/dL)	83 ± 5	81 ± 5	81 ± 6	76 ± 12	ns
Uric acid (mg/dL)	4.05 ± 0.89	3.20 ± 0.67^f^	3.60 ± 0.57	3.90 ± 1.02^w^	W*F (*P* = 0.028)
Creatinine (mg/dL)	0.30 ± 0.40	0.33 ± 0.06	0.34 ± 0.08	0.33 ± 0.05	ns
Triglycerides (mg/dL)	73 ± 18	59 ± 14	74 ± 47	60 ± 6	ns
Total cholest. (mg/dL)	166 ± 28	174 ± 20	170 ± 33	162 ± 23	ns
HDL cholest. (mg/dL)	46 ± 8	59 ± 11^f^	60 ± 16^w^	51 ± 9	W*F (*P* = 0.006)
LDL cholest. (mg/dL)	106 ± 26	104 ± 20	96 ± 27	102 ± 18	ns
Insulin (µIU/mL)	5.88 ± 1.68	4.24 ± 2.25	6.75 ± 3.71	4.06 ± 2.25	F (*P* = 0.017)
HOMA-index	1.21 ± 0.39	0.85 ± 0.47	1.31 ± 0.66	0.78 ± 0.44	F (*P* = 0.012)

Results are mean ± standard deviation (SD). Statistical analysis: W: effect of birth weight; F: effect of the type of infant feeding; W*F: interactive effect between birth weight and type of infant feeding (*P* < 0.05, two-way ANOVA). f represents differences between FF and BF within the same group of birth weight; w represents differences between AGA and SGA within the same group of infant feeding (*P* < 0.05, Student’s t test)

AGA, appropriate for gestational age; SGA, small for gestational age; BF, breastfed; FF, formula fed; SBP, systolic blood pressure; DBP, diastolic blood pressure; HR, heart rate; BMI, body mass index; HDL, high-density lipoprotein; LDL, low-density lipoprotein; HOMA, homeostasis model assessment; ns, not-significant

At 5 years, all children had normal biochemical values for their age, and no significant differences between groups were found except for insulin and HOMA index, which were significantly lower in FF vs BF children (*P *= 0.017 and *P *= 0.012, respectively; two-way ANOVA). An interactive effect was also found by two-way ANOVA regarding uric acid and HDL-cholesterol between type of feeding and BW at birth (*P *= 0.028 and *P *= 0.006, respectively). Specifically, FF children of the SGA displayed higher uric acid levels than FF children of the AGA group (*P *= 0.048, Student’s t test); in addition, in the AGA group, uric acid levels were lower in FF subjects compared with BF subjects (*P *= 0.027, Student’s t test). Regarding HDL-cholesterol, BF subjects of the SGA group displayed higher levels than BF subjects of the AGA group (*P *= 0.045, Student’s t test); in addition, FF subjects of the AGA group showed higher HDL-cholesterol than BF subjects (*P *= 0.011, Student’s t test).

### Gene expression levels in peripheral blood cells

Gene expression analysis in PBC was performed in children at 5 years. Blood samples for gene expression were available from 38 children out of the 40 that participated in the study.

**Fig. 1:**
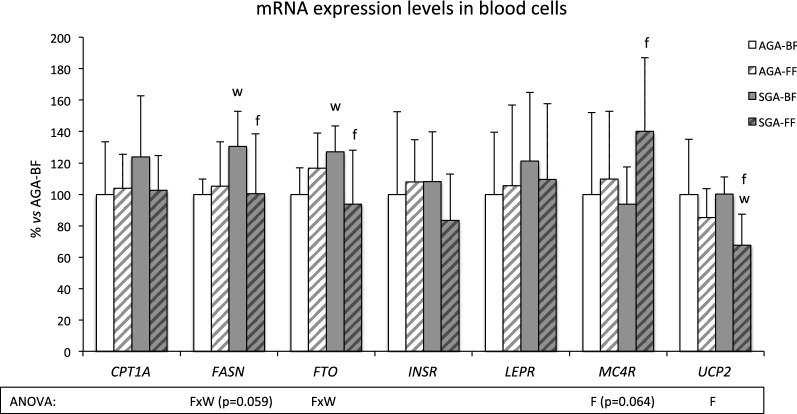
Gene expression data. Transcript levels in peripheral blood cells of CPT1A, FASN, FTO, INSR, LEPR, MC4R and UCP2 at 5 years in breastfed (BF) or formula-fed (FF) children whose birth weight was appropriate or small for gestational age (AGA or SGA). Results are mean ± SD. Statistical analysis: F: effect of the type of infant feeding; W*F: interactive effects between birth weight and the type of infant feeding (P < 0.05, two-way ANOVA). f represents differences between FF and BF within the same group of birth weight; w represents differences between AGA and SGA within the same group of infant feeding (P˂ 0.05, Student’s t test)

Results showing expression levels of selected genes in PBC at 5 years in AGA and SGA children, taking into account if they were breastfed or formula-fed, are shown in Fig. 1. Results showed that expression levels of *UCP2* were significantly lower in FF children compared with BF ones (*P *= 0.002, two-way ANOVA), and differences were more marked in SGA children (*P *< 0.001, Student’s t-test). Moreover, FF children of the SGA group displayed lower expression of *UCP2* than those of the AGA group (*P *= 0.054, Student’s t-test).

An effect of the type of infant feeding was also found regarding *MC4R*. Expression levels were higher in FF subjects compared with BF ones, although differences did not reach statistical significance (*P *= 0.064, two-way ANOVA). However, differences were more marked among SGA children, since those that were FF showed higher expression levels of MC4R than BF children (*P *= 0.017, Student’s t-test).

Concerning *FASN* and *FTO* transcripts, there was an interaction between type of infant feeding and BW at birth (*P *= 0.059 and *P *= 0.003, respectively, two-way ANOVA). Among SGA children, BF ones displayed higher expression levels of both genes than FF ones (*P *= 0.047 and *P* = 0.014, respectively, Student’s t-test). In addition, among BF children, those that were SGA displayed higher expression levels of both genes than those that showed AGA (*P *= 0.004 and *P *= 0.005, respectively, Student’s t-test).

No significant differences were found regarding transcript levels of *LEPR*, *INSR*, or *CPT1A* concerning BW at birth or type of infant feeding.

### Association of gene expression on anthropometric, blood pressure and biochemical parameters of subjects

The potential association between anthropometric, blood pressure and metabolic parameters at 5 years of age, and gene expression at this age was analysed using a multiple regression analysis, which included each parameter as the dependent variable and gene expression as independent variable (Table 2). No significant associations were found between gene expression and anthropometric measurements (data not shown). Nevertheless, in terms of blood pressure, systolic blood pressure (SBP) was negatively associated with *INSR* expression levels (*P *= 0.018) (Table 2). Regarding metabolic parameters, uric acid levels were negatively associated with the expression levels of *UCP2* (*P *= 0.031); HDL-cholesterol levels were negatively associated with expression levels of *FASN* (*P *= 0.022) and positively with expression levels of *FTO* (*P *= 0.007); and insulin levels were positively associated with expression levels of *FASN* (*P *= 0.011) (Table 2).

**Table 2:** Association between office systolic BP, metabolic parameters and gene expression at 5 years of age estimated by multiple regression analysis

Variable	Beta	*P* value
Systolic blood pressure		
*CPTL1*	0.161	0.473
*FASN*	0.133	0.578
*FTO*	0.199	0.398
*INSR*	*− 0.534*	*0.018*
*LEPR*	*− *0.128	0.578
*MC4R*	*− *0.031	0.903
*UCP2*	*− *0.003	0.989
R^2^: 0.279		
Uric acid		
*CPTL1*	0.393	0.138
*FASN*	*− *0.109	0.690
*FTO*	*− *0.128	0.558
*INSR*	*− *0.026	0.910
*LEPR*	*− *0.096	0.692
*MC4R*	*− *0.122	0.668
*UCP2*	*− 0.546*	*0.031*
R^2^: 0.38		
HDL cholesterol		
*CPTL1*	0.409	0.071
*FASN*	*− 0.534*	*0.022*
*FTO*	*0.590*	*0.007*
*INSR*	0.126	0.525
*LEPR*	0.152	0.476
*UCP2*	0.121	0.559
*MC4R*	0.176	0.474
R^2^: 0.49		
Insulin		
*CPTL1*	*− *0.115	0.598
*FASN*	*0.672*	*0.011*
*FTO*	*− *0.170	0.451
*INSR*	*− *0.414	0.062
*LEPR*	*− *0.086	0.689
*MC4R*	0.126	0.605
UCP2	0.205	0.381
R^2^: 0.337		

Data with significant *P* value (< 0.05) are indicated in italic

*CPT1A*, carnitine palmitoyl transferase 1 alpha; *FASN*, fatty acid synthase; *FTO*, alpha-Ketoglutarate dependent dioxygenase; *INSR*, insulin receptor; *LEPR*, Leptin receptor, long-form; *MC4R*, Melanocortin 4 receptor, *UCP2*, Uncoupling protein 2

## Discussion

The present prospective study performed in children born at term after a non-complicated pregnancy shows significant differences in transcript levels in PBC of genes related to different metabolic pathways at the age of 5 years, according to birthweight and the type of infant feeding. Notably, expression levels were particularly different as an effect of the type of infant feeding in SGA children. These results are in agreement with the potential interest of gene expression levels in blood cells at early ages as a marker of metabolic programming effects associated with birth weight and the type of infant feeding, which may thereby predict the susceptibility to metabolic alterations in later life. In contrast, no relevant differences in body weight, metabolic profile or BP were found at 5 years of age, with the exception of uric acid, HDL-cholesterol and insulin levels. Nevertheless, in all cases, values of these parameters were in the normal range for age.

Regarding gene expression, one of the most significant results of the present study is the different expression of *UCP2* in PBC in terms of type of infant feeding. *UCP2* was downregulated in FF children compared with BF ones, and the differences were more marked among the SGA subjects. Differences in *UCP2* expression levels according to the type of infant feeding have been previously described in a group of 237 children aged 2–9 years from eight European countries [22]. We previously described that breastfed male subjects showed higher expression levels of this gene compared with formula-fed ones [22]. Notably, our present results, considering both male and female, confirm those findings. In addition, these results also show the importance of BF among children in the SGA group, since it normalizes *UCP2* expression levels to those of the AGA + BF group, which could be considered as the reference group in terms of future metabolic health.

UCP2 is involved in a broad range of pathological processes. Remarkably, we have found an inverse association between *UCP2* expression levels in PBC and uric acid levels. This relationship can be explained by the function of UCP2. This protein is present in the inner mitochondrial membrane and it mainly acts in the protection against oxidative stress, decreasing the ATP levels and reactive oxygen species (ROS) produced by electron transport [39]. Uric acid overproduction may occur as a result of the acceleration of ATP degradation to AMP, a precursor of uric acid [40] and UCP2 could decrease the ATP level and thereby reduce AMP levels for uric acid formation [41]. Therefore, although a cause-effect relationship cannot be established from our results, the presence of increased *UCP2* expression levels as mirrored in PBC might reflect an increased potential to regulate ROS and the ATP/AMP ratio, and thereby a decreased risk of hyperuricemia and other related metabolic complications. The inverse association between *UCP2* expression levels in PBC and uric acid levels is also of interest regarding the relevance of uric acid levels in relation to cardiometabolic risk factors, particularly high blood pressure and elevated insulin and triglycerides [42]. Lurbe et al. have previously described that uric acid levels in children at 5 and 10 years were significantly and independently associated to birth weight [23, 43], and they were also positively correlated with blood pressure, insulin and triglycerides in overweight and obese youths [42].

Other signs on the potential connection between *UCP2* expression and uric acid levels have been previously suggested from studies in adult Chinese population showing a relation between the risk of hyperuricemia and genetic variants, such as the − 866G/A (rs659366) polymorphism located in the promoter region of *UCP2* [41]. Females with the A allele, which is associated with higher *UCP2* mRNA expression, had lower serum urate and a decreased risk of hyperuricemia, but no relation was found in males [41]. Therefore, the relationship between transcript levels of *UCP2* in PBC and uric acid levels in blood merits further consideration in studies regarding early prevention of cardiometabolic risk factors.

MC4R has a well-established role in feeding behavior and energy homeostasis [44]. Transcript levels of *MC4R* were higher in FF children compared with BF children, particularly in SGA infants. It is noticeable that, as mentioned above for *UCP2*, breastfeeding normalized expression levels of *MC4R* in SGA children to the “reference” levels of the AGA + BF group. The meaning of differences in the expression levels of *MC4R* in PBC is not known, and, to our knowledge, this is the first study addressing *MC4R* expression levels in PBC in relation to birth weight and type of infant feeding. In a previous study, high expression levels of this gene in PBC were associated with the intake of high percentage of energy from fat, but also with lower body fat percentage and BMI z-score in children [45]. Here, although dietary intake was not assessed, no differences were found in terms of anthropometric parameters between BF and FF children of the SGA group, and no significant associations were found between expression levels of this gene and anthropometric and biochemical measurements performed at 5 years of age. However, the small sample size could be the reason for the lack of correlations. In any case, present findings suggest that greater *MC4R* expression levels in PBC in children may be indicative of adverse programming events during intrauterine life, while normalization of transcript levels of this gene in children who were SGA may be indicative of adequate phenotypic reprogramming by adequate nutritional supply during lactation. Notably, considering the inverse trends between the expression levels of *MC4R* and *UCP2* in SGA children according to the type of infant feeding, besides the potential interest of individual gene expression values by itself, the *MC4R*/*UCP2* mRNA ratio could be of greater relevance as a potential, more sensitive marker of the beneficial effects of breast feeding in SGA infants.

Transcript levels of *FASN* in PBC have also been raised as potential biomarkers of metabolic alterations, particularly related with lipid metabolism. Specifically, in children, upregulation of *FASN* expression in PBC has been associated with lower triglyceride levels [21]. Moreover, *FASN* transcript levels in PBC have also been reported to be higher in children who were BF compared with FF ones [22]. Here, no relationship has been found between expression levels of this gene and triglyceride levels at 5 years of age (data not shown), which could be tentatively attributed to the limited number of subjects. Although triglycerides were within the normal range and no differences were found between groups. Nevertheless, the presence of higher *FASN* expression levels in BF compared with FF children, but only in SGA subjects, is also described here, in agreement with previous results [22]. Notably, association studies have revealed that *FASN* expression levels in PBC are positively related with insulin levels at 5 years of age, which were found to be higher in BF compared with FF children and the difference was apparently greater in children that were SGA at birth. The biological meaning of this association could be interpreted according to the known function of insulin activating *FASN* expression [46], and the fact that PBC may reflect changes in gene expression occurring in different tissues, such as liver, which has been described in animal models [20]. On the other hand, the negative association found between *FASN* expression levels and HDL-cholesterol is more difficult to interpret, but it could be tentatively related with the essential role of insulin regulating HDL-cholesterol metabolism, since it favors HDL2 to HDL3 conversion, through its action on hepatic lipase [47]. However, in both cases, the possible clinical interest of these associations, if any, remains to be established.

A similar pattern to that of *FASN* has been observed regarding *FTO* expression. Breastfeeding was associated with *FTO* upregulation in PBC, but only in the SGA group. This fact also supports the hypothesis that the benefits of breastfeeding could be even more important in SGA subjects, and that such transcript-based biomarkers could reflect these early events more accurately than other clinical markers.

Despite the recognized relevance of *FTO* gene variants on obesity predisposition [33, 34] the relation between *FTO* expression in blood cells and biochemical or clinical parameters related to metabolic syndrome is not well-defined. In adult humans, higher mRNA expression levels of *FTO* have been described to be greater in adipose tissue of obese than normal weight individuals, but no differences have been found regarding *FTO* expression in peripheral blood mononuclear cells (PBMC) [48]. Here, we have found a positive correlation between *FTO* expression levels in PBC and HDL-cholesterol at 5 years of age. Evidence on the relationship between FTO and cardiovascular disease (CVD) exists from studies showing the association of *FTO* gene variants with CVD risk, independently of BMI [49]. Some studies have described an association between the FTO gene rs9939609 polymorphism and HDL-cholesterol. Individuals carrying the risk allele, displayed decreased HDL-cholesterol concentration [50]. Nevertheless, whether or not the association found in the present study involves causality remains to be ascertained.

Unlike the aforementioned genes, expression levels of other candidate genes studied in the present study, *CPT1L, INSR and LEPR* were not significantly different between groups. Results regarding *CPT1L* and *LEPR* agree with previous published results showing no significant differences in their transcript levels in PBC between BF and FF children [22]. However, increased expression levels of *INSR* have been previously described in BF children compared with FF children [22]. Here, no differences have been found in AGA children, but BF children from the SGA group showed a non-significant trend to higher *INSR* expression levels compared to FF children (*P *= 0.098). The limited sample size could be the reason for the lack of statistical significance. This is in accordance with the general pattern described for other genes studied. Moreover, the finding of a negative correlation between transcript levels of *INSR* and SBP at 5 years of age may be considered in the light of the known link between insulin resistance and hypertension [51, 52]. In this regard, it is plausible to consider changes in the expression levels of *INSR* in PBC as an early molecular marker of future alterations frequently associated with hypertension.

The strengths and limitations of the present study must be taken into account. The study has a prospective design, with subjects recruited from birth, and includes data on pregnancy, birth, and early childhood growth, together with BP values and metabolic data, as well as data on gene expression analysis in blood cells. The study is focusing on an area with a lack of research: The relationship between BW and type of infant feeding, with gene expression in PBC as potential biomarkers of future metabolic status. However, one of the limitations of the study is the small sample size, which reduces statistical power. Another limitation may be the use of insulin levels and HOMA index as surrogate markers of insulin resistance, which differs from the gold standard method, the hyperinsulinemic euglycemic clamp [53]. Nevertheless, the use of this method is mainly restricted to investigational facilities and is normally unavailable in the clinical setting.

## Conclusions

In summary, present results show differences in gene expression in PBC in 5-year-old children due to the type of infant feeding, which, interestingly, are more marked in infants born SGA compared with those in the AGA group. This is particularly evident for *MC4R* and *UCP2*, whose expression levels were increased and decreased, respectively, in FF children and especially in SGA, which may be indicative of adverse programming during intrauterine life. Therefore, although it needs to be independently confirmed in future studies, increased *MC4R*/*UCP2* mRNA ratio in PBC may be a hallmark of FF infants born SGA; whereas normalization of this ratio could be indicative of adequate reprogramming by appropriate nutrition during lactation. Results also show differences in *FASN* and *FTO* expression levels in PBC due to the type of infant feeding that were observed only in SGA children. The finding of a negative correlation between transcript levels of *UCP2* and uric acid levels is also relevant, considering the relation of uric acid with cardiometabolic risk factors, such as blood pressure and circulating insulin and triglycerides [42]. Therefore, interventions to avoid the elevation of uric acid levels should be considered and breast feeding might play an important role. Overall, these results suggest that SGA infants are more sensitive to the type of feeding during lactation and gene expression of particular genes in PBC may precisely reflect these effects in the absence of clear differences in phenotypic traits.

In terms of future work, it would be interesting to increase the sample size and power of analysis to be able to validate and make further correlations between gene expression and phenotypes and risk factors.

## Abbreviations

AGA: adequate for gestational age
AMP: adenosine monophosphate
ATP: adenosine triphosphate
BF: breastfed
BMI: body mass index
BMIz: BMI z-score
BP: blood pressure
BW: body weight
cm: centimeters
CPTA1: carnitine palmitoyl transferase 1 alpha
Ct: threshold cycle
CVD: cardiovascular disease
FASN: fatty acid synthase
FF: formula-fed group
FTO: alpha-Ketoglutarate dependent dioxygenase
g: grams
HDL: high-density lipoprotein
HOMA: homeostatic model assessment
HR: heart rate
INSR: insulin receptor
kg: kilograms
L: lambda
LDL: low-density lipoprotein
LEPR: leptin receptor, long-form
M: Mu
MC4R: melanocortin 4 receptor
PBC: peripheral blood cells
PBMC: peripheral blood mononuclear cells
PCR: polymerase chain reaction
RNA: ribonucleic acid
ROS: reactive oxygen species
S: sigma
SBP: systolic blood pressure
SD: standard deviation
SGA: small for gestation age
TRIM27: homo sapiens tripartite motif containing 27
UCP2: uncoupling protein 2
WHO: World Health Organization
